# Comparative evaluation of involved free light chain and monoclonal spike as markers for progression from monoclonal gammopathy of undetermined significance to multiple myeloma

**DOI:** 10.1002/ajh.25999

**Published:** 2020-09-29

**Authors:** Charlotte Gran, Johan Liwing, Arnika Kathleen Wagner, Andre Verhoek, Ana Gezin, Evren Alici, Hareth Nahi

**Affiliations:** ^1^ Department of Medicine Karolinska Institutet Stockholm Sweden; ^2^ Department of Clinical Chemistry Karolinska University Laboratory Stockholm Sweden; ^3^ Ingress‐Health Nederland BV Rotterdam The Netherlands; ^4^ Haematology Center Karolinska University Hospital Stockholm Sweden

## Abstract

Monoclonal gammopathy of undetermined significance (MGUS) is a premalignant clonal plasma cell disorder, with a 1% yearly risk of progression to multiple myeloma (MM). Evolution of M‐spike and serum free light chain (sFLC) during follow‐up could identify patients at high risk of progression. In this region‐wide study, including 4756 individuals, 987 patients with MGUS were identified, and baseline factors as well as evolving involved FLC (iFLC) were evaluated as potential markers for risk of progression from MGUS to MM. Furthermore, evolving iFLC and M‐spike were assessed quarterly for a median of 5 years. At baseline, patients that progressed had significantly higher iFLC compared to non‐progressors. The risk factors of M‐spike >1.5 g/dL, age >65 years and iFLC >100 mg/L were all independently associated with increased risk of MGUS to MM progression. For patients that had any two or three risk factors, the 5‐year cumulative probability of progression was significantly higher (31%) compared to no risk factors (2%). Evolving iFLC >100 mg/L during follow‐up was consistently associated with increased risk of progression. Based on our observations, we propose to include iFLC as a monitoring tool for all MGUS patients. Furthermore, we recommend a quarterly monitoring in all high‐risk patients. Finally, we suggest that the risk of MGUS progression should be stratified with age, M‐spike, and iFLC at baseline.

## 1 INTRODUCTION

Monoclonal gammopathy of undetermined significance (MGUS) is currently defined as a premalignant clonal plasma cell disorder characterized by production of a serum monoclonal protein. It is observed in 3.2% of those aged >50 years^1^ and is diagnosed incidentally in routine laboratory tests. It is considered to be a precursor‐stage of multiple myeloma (MM), and in 1% of patients per year,^2^ progresses to MM, but also to other plasma cell disorders such as light‐chain amyloidosis or other lymphoproliferative diseases including Waldenstrom's macroglobulinemia.

Monoclonal gammopathy of undetermined significance often precedes MM with a constant annual risk, thus, life‐long follow‐up is necessary.^3^, ^4^ Several risk factors associated with progression to MM have been suggested, with a non‐IgG M‐protein,^5^, ^6^ M‐spike >1.5 g/dL^5^, ^6^ and abnormal serum free light chain (sFLC) ratios (FLCr)^6^ incorporated into current International Myeloma Working Group (IMWG) risk criteria.^2^ Evolving M‐protein (increasing levels over time) has been suggested as a risk factor in smoldering multiple myeloma (SMM) in smaller retrospective studies.^7^, ^8^, ^9^ Furthermore, it has been reported that MGUS patients that progress to MM convert from low/intermediate‐risk to high‐risk MGUS prior to the MM diagnosis.^10^

Abnormal FLCr are detected in more than 95% of MM but only in 30%‐47% of MGUS cases.^4^, ^6^, ^11^, ^12^, ^13^ It can be hypothesized that evolving sFLC could be a predictor for progression from MGUS to MM. The current IMWG diagnosis criteria recommend measuring sFLC in combination with serum protein electrophoresis (sPEP) and immunofixation when screening for plasma cell dyscrasia and for risk classifications. However, sFLC measurements are not currently included in MGUS monitoring guidelines.^2^

It is of importance to identify high‐risk MGUS groups, both for better risk classification at diagnosis in conjunction with a more optimal monitoring during follow‐up. Furthermore, high‐risk MGUS patients may potentially be considered for preventive measures to limit the progression to MM. As the percentage of patients with detectable iFLC are, and becoming higher in MM compared to MGUS, we hypothesized that evolving iFLC during monitoring may be an essential risk factor for progression. We therefore analyzed a unique dataset from hematology clinics and primary care units in Sweden, with up to 46 years of follow‐up.

## 2 METHODS

### 2.1 Study population

The study proposal was approved by the Swedish Ethical Review Authority (EPN: 2017/349‐31) and was performed in accordance with the Helsinki declaration.

For this study, we queried the Karolinska University Laboratories database, which constitutes data from 20% of the Swedish population, to identify individuals with sFLC measurements from 1 September 2009, until 1 September 2017. All individuals older than 18 years, with an analysis of serum or urine PEP (s/uPEP) within 7 days of sFLC measurement were included. In total, 4756 individuals were included, which generated 30 052 sampling occasions from all individuals during the study period. The electronic medical records for all patients included in the study were reviewed for records of MGUS and MM diagnosis date as well as to be able to exclude patients with other plasma cell disorders or hematological disorders. So, 2003 individuals that had no plasma cell disorders or evidence of M‐protein in s/uPEP or abnormal FLCr were excluded. And, 1766 patients were excluded due to diagnoses of either plasma cell disorders or hematological diseases other than MGUS. Upon further exclusion of the IgM MGUS population, the remaining study population consisted of 987 patients with an MGUS diagnosis of either IgG, IgA, IgD or light‐chain type (Figure S1). All MGUS patients were required to have no CRAB symptoms (hypercalcemia, renal failure, anemia and bone lesions) and a M‐protein value equal to or below 3.0 g/dL.^2^ Patients with high risk MGUS or symptoms of bone lesions were referred to imaging according to local guidelines. In patients with a M‐protein equal to or below 1.5 g/dL (n = 836) a confirmatory bone marrow was not required for MGUS diagnosis. In the remaining 151 patients, a bone marrow confirmation of the MGUS diagnosis were only obtained in 73 patients.

For each individual, besides sFLC, routine laboratory and clinical parameters were collected and compiled from all available sampling occasions during the study period (Table 1).

**TABLE 1:** Patient characteristics and univariate associations with the risk of progression from MGUS to MM

	MGUS‐NP (N = 904)	MGUS‐MM (N = 83)	*P* value	Hazard Ratio for Risk of Progression^a^	*P* value	Five Year Cumulative Probability of Progression	*P* value
	No. patients (%)			(95% CI)		% (95% CI)	
Patient‐relatable variable							
Gender, male no. (%)	494 (55)	38 (46)	.21	0.93 (0.60‐1.43)	.73		
Age at MGUS diagnosis, years							
Age, median (range)	69 (26‐96)	66 (35‐86)	.51	1.03 (1.01‐1.05)	**.004**		
18‐65	348 (39)	33 (40)	.80	1.00	**.002**	4 (2‐7)	.98
>65	549 (61)	49 (60)		2.1 (1.31‐2.40)		9 (7‐13)	
**Disease‐relatable Variable**	**Median (IQR)**						
Hemoglobin, g/dL	129 (118‐140)	128 (115‐137)	.36	0.97 (0.97‐1.00)	**.04**		
Creatinine, μmol/L	83 (68‐110)	78 (66‐103)	.56	1.0 (0.99‐1.02)	.52		
eGFR, mL/min/1,73 m^2^	65 (47‐77)	66 (52‐78)	.66	1.0 (0.99‐1.01)	.54		
Calcium, mmol/L	2.3 (2.2‐2.4)	2.3 (2.2‐2.4)	.96	1.1 (0.17‐7.28)	.92		
Plasma M‐spike, g/dL	0.6 (0.1‐1.1)	1.6 (0.7‐2.5)	**<.001**	1.08 (1.00‐1.16)	**.048**		
Urine M‐spike, mg/L	7 (4‐20)	8 (0‐31)	**<.001**	1.02 (0.97‐1,06)	.47		
Involved FLC, mg/L	10 (0‐30)	43 (10‐219)	**<.001**	1.19 (1.10‐1.29)	**<.001**		
	**no. patients (%)**						
Stage ISS at MGUS diagnosis, no. (%)							
III	42 (8)	5 (14)	.43	1.00	.10	24 (10‐52)	.03
I + II	477 (92)	31 (86)		0.45 (0.14‐2.59)		5 (4‐8)	
Heavy chain type							
IgG	572 (72)	55 (69)	.54	1.00	.35	6 (4‐9)	.45
Non‐IgG	227 (28)	25 (31)		1.25 (0.78‐2.02)		10 (6‐15)	
Plasma M‐spike							
≤1.5 g/dL	795 (88)	41 (49)	**<.001**	1.00	**<.001**	4 (2‐9)	**<.001**
>1.5 g/dL	108 (12)	42 (51)		1.84 (0.91‐3.69)		7 (6‐10)	
iFLC			**<.001**		**<.001**		**<.001**
≤100 mg/L	806 (89)	56 (67)		1.00		5 (4‐7)	
>100 mg/L	98 (11)	27 (33)		4.22 (2.66‐6.70)		19 (12‐29)	
sFLC ratio			**<.001**		**<.001**		**<.001**
Normal sFLC ratio	563 (62)	27 (32)		1.00		4 (3‐7)	
Abnormal sFLC ratio	341 (38)	56 (68)		2.44 (1.53‐3.88)		10 (7‐14)	

*Note*: All statistically significant values are provided in bold. MGUS‐NP denotes MGUS patients that does not progress to MM, MGUS‐MM MGUS patients that progress to MM, M‐spike concentration of M‐component, iFLC involved FLC levels, sFLC serum FLC, and ISS Multiple Myeloma International Staging System.

^a^ The hazard ratio refers to the risk of progression to MM by each variable. The variables are both evaluated as continuous variables (hemoglobin, creatinine, eGFR, calcium, plasma and urine m‐spike and iFLC) and as categorical variables (ISS, heavy chain type, m‐spike>1.5 g/dL, iFLC > 100 mg/L and abnormal sFLC ratio).

Regarding s/uPEP; M‐protein of heavy and light chain types, M‐protein size, total immunoglobulin levels, urine kappa and lambda levels, were included in the database. Serum FLC assays were conducted with latex‐enhanced immunonephelometric assay (Siemens Healthcare GmbH, Erlangen, Germany). Total serum immunoglobulin (IgG, IgA, and IgM) concentrations were analyzed using immunoturbidimetric assay (Roche Diagnostics GmbH, Mannheim, Germany). Urine light chains (kappa and lambda) concentrations were analyzed using immunonephelometric assay (Siemens Healthcare GmbH, Erlangen, Germany). S/uPEP and immunofixation were performed with agarose gels on the Hydrasys/Hydrasys 2 platform (Sebia, Lisses, France).

Sequential data were evaluated in a subgroup of the main MGUS cohort, where at least two serial samples, for example, a baseline sample and one additional sample, with a minimum of 3 months between, per patient were available during the study period and before the progression to MM, and where the sample nearest to MM diagnosis was stipulated to be a minimum of 6 months prior to the date of the diagnosis. The sequential cohort (n = 516), comprised a total of 5364 sampling occasions for all MGUS patients, median serial samples four (range 2‐56). In total, 141 patients had the minimum of two serial samples and 375 patients had three or more serial samples. In patients progressing from MGUS to MM, serial samples before the MM diagnosis were selected, resulting in 44 patients (9%) with MGUS developing MM during the follow‐up time.

### 2.2 Statistical analyses

The endpoint was time from first sample available at MGUS diagnosis (baseline sample) to progression of MM, either smoldering or treatment demanding MM as defined by IMWG.^14^

The effects of prognostic factors were estimated by univariate Cox regression, where the hazard ratios (HR) (CI threshold: 95% and *P* value threshold <.05) were reported. Log‐rank tests were performed for inter‐group comparisons. The Kaplan‐Meier method was used to calculate medians at 60 months and their associated 95% confidence intervals.

Multivariate analyses, including all variables significant (*P* < .05) in the univariate regressions using a backward selection, were assessed with Cox proportional hazard regression. Similar to the univariate regressions, the time to event is defined as the time from the first sample available at MGUS diagnosis to the end of follow‐up or progression to MM. Both univariate and multivariate analysis were performed with continuous values to evaluate the effect of subsequent increasing values, where the log value was taken of the plasma/urine M‐spike and the iFLC to better handle extreme values, as well as categorical variables to evaluate the effect of the cut‐off values for risk factors.

For sequential samples, the increase in M‐spike and involved FLC (iFLC) of 0.5 g/dL and 100 mg/L were chosen according to the IMWG MM progression criteria.^15^, ^16^ We analyzed the absolute threshold and absolute change for each variable in a defined time interval (3 months). The thresholds were defined as 100 mg/L for iFLC and 0.5 g/dL for M‐spike. The absolute thresholds were implemented for each individual patient and variable, every third month to be either above (event) or below (non‐event) the threshold values, independent of the baseline value. The absolute changes were defined as the difference between the baseline value and the subsequent values, measured every third month for each individual patient and variable. An increase of >100 mg/L for iFLC and/or >0.5 g/dL for M‐spike in the subsequent values were defined as an event, per each variable. The HR for change in absolute threshold and absolute change for each variable for each 3 month interval were estimated by univariate Cox regression.

For the selected indicators, time threshold and prognostic variables receiver operating characteristic (ROC) method was used to evaluate the prediction reliability, specificity and sensitivity.

## 3 RESULTS

### 3.1 Patient characteristics

The main MGUS cohort included 987 MGUS patients, 530 (54%) were male, and the median age was 68 years (range 26‐95 years). The median follow‐up time was 64 months, 5824 person‐years. There were 904 (92%) patients with MGUS that did not progress to MM (MGUS‐NP), while 83 (8%) patients with MGUS developed MM (MGUS‐MM) during the follow‐up time (Table 1). A sub‐analysis of light chain MGUS (LCMGUS) was performed, including in total 89 patients were included in the cohort out of which seven (8%) progressed to MM.

No significant difference between MGUS‐NP and MGUS‐MM were observed with regards to gender, age, hemoglobin, creatinine, calcium or eGFR at baseline. Cytogenetics by FISH was available in 7% of the MGUS population. Due to the low number of patients with available data, no further analysis could be reliably performed on this specific population. During the follow‐up, progression to MM occurred in 18 of 73 patients (25%) with available bone marrow assessment compared to 20 out of 78 patients (26%) in patients with M‐spike >1.5 g/dL without available bone marrow assessment. In the main cohort, baseline values of abnormal FLCr were observed in 56 (68%) MGUS‐MM patients compared to 339 (38%) MGUS‐NP (*P* < .001). Note, M‐spike >1.5 g/dL at baseline was more frequent in MGUS‐MM (n = 42, 51%) compared to MGUS‐NP (n = 108, 12%, *P* = .003). No significant differences between MGUS‐MM and MGUS‐NP in heavy chain types were observed (Table 1).

In the sequential cohort, M‐spike >1.5 g/dL and abnormal FLCr were observed more frequently in MGUS‐MM (*P* < .001) compared to MGUS‐NP (*P* < .001). However, no differences were observed for heavy chain type between MGUS‐MM and MGUS‐NP (Table S1).

### 3.2 Risk of progression

The five‐year cumulative probability of progression was 5% for the whole MGUS cohort (Figure S2) which is in line with previously published reports.^5^, ^17^

### 3.3 By IMWG classification

Previously reported factors associated with progression from MGUS to MM are shown in Table 1. Of these risk factors, abnormal FLCr (*P* < .001) and serum M‐spike >1.5 g/dL (*P* < .001) were associated with increased risk of progression to MM, whereas non‐IgG was not associated with any increased risk.

### 3.4 Proposed additional factors at diagnosis

Age was a major correlate to progression, both as a continuous variable and with cut‐offs of >65 years and >70 years. The yearly HR for age as a continuous variable was 1.03 (95% CI: 1.01‐1.95, *P* = .004). In patients with the age >65 years at MGUS diagnosis, the risk of progression to MM was higher compared to patients <65 years of age, (HR: 2.1, 95% CI: 1.31‐2.40, *P* = .002), and (HR: 1.90, 95% CI: 1.18‐3.05, *P* = .008) for patients >70 vs <70 years.

The iFLC concentration correlated with increased risk of progression to MM (HR: 1.08, 95% CI: 1.00‐1.16, *P* = .048). Furthermore, iFLC >100 mg/L at baseline strongly correlated to MM progression, (HR: 4.22, 95% CI: 2.66‐6.70, *P* < .001). As an abnormal FLCr can be anticipated when iFLC levels exceed 100 mg/L, a sub analysis of FLCr as a risk factor in patients with iFLC <100 mg/L was performed. A significant increased risk of progression was observed also in these patients, HR: 2.27, 95% CI: 1.34‐3.85, *P* = .002.

A separate univariate analysis of the LCMGUS cohort including the risk factors (iFLC >100 mg/L, abnormal FLCr and >65 years) showed an increased risk of progression in patients with iFLC >100 mg/L (HR:5.98, 95% CI: 1.16‐30.9, *P* = .033) while no significance was observed for either age >65 or, interestingly, FLCr. The lack of significance in the later factors is likely affected by the low numbers of patients with LCMGUS.

### 3.5 Multivariate analysis

Significant factors for progression from the univariate analyses were further studied by multivariate analyses. In the multivariate analysis for continuous variables, significance was retained for log (iFLC) concentrations (HR: 1.18, 95% CI: 1.09‐1.28, *P* < .001). For categorical multivariate analysis, age >65 and iFLC >100 mg/L remained significant. While no significance was observed for M‐spike as a continuous variable, when investigating it with the cut‐off reported by IMWG, >1.5 g/dL, the factor was significant, *P* < .001 (Table 2).

**TABLE 2:** Multivariate associations with the risk of progression from MGUS to MM

	Hazard ratio for risk of progression	*P* value
	(95% CI)	
MVA continuous variables		
Hemoglobin	0.99 (0.97‐0.99)	**.029**
Log plasma M‐spike	1.07 (0.99‐1.16)	.089
Log iFLC	1.18 (1.09‐1.28)	**<.001**
MVA categorical variables		
Plasma M‐spike		
≤1.5 g/dL	1.00	
>1.5 g/dL	3.23 (1.95‐5.33)	**<.001**
iFLC		
≤100 mg/L	1.00	**.008**
>100 mg/L	2.35 (1.25‐4.40)	
LCr		
Normal FLCr	1.00	.43
Abnormal FLCr	1.26 (0.71‐2.24)	
Age at MGUS diagnosis		
18‐65	1.00	**.003**
>65	2.21 (1.30‐3.74)	
MVA categorical variables with iFLC and FLCr combined		
Plasma M‐spike		
≤1.5 g/dL	1.00	
>1.5 g/dL	3.24 (1.96‐5.35)	**<.001**
iFLC/FLCr		
iFLC ≤100 mg/L and normal FLCr	1.00	
iFLC ≤100 mg/L and abnormal FLCr	1.24 (0.68‐2.22)	.46
iFLC >100 mg/L	2.93 (1.57‐5.47)	**.001**
Age at MGUS diagnosis		
18‐65	1.00	**.003**
>65	2.21 (1.30‐3.74)	

*Note*: All statistically significant values are provided in bold. Three separate MVAs were performed, risk factors significant in univariate analysis where entered either as continuous or categorical variables. iFLC denotes involved free light chain, FLCr serum free light chain ratio.

We further assessed the impact of risk of progression to MM with iFLC above or below 100 mg/L separate from the FLCr. The patients were stratified as follows; patients with iFLC <100 and normal FLCr as reference, patients with iFLC <100 and abnormal FLCr as group one and patients with iFLC >100 (regardless of FLCr results) as group two, thus excluding FLCr for patients with iFLC >100. This combined parameter was entered into multivariate analysis together with age >65 years and M‐spike >1.5 g/dL. The HR for iFLC >100 mg/L, regardless of FLCr, for this model was higher compared to the model including M‐spike >1.5 g/dL, age >65, FLCr abnormal and >100 mg/L (Table 2).

Another factor that was significant in univariate/multivariate analyses was hemoglobin. However, when investigating the differences between MGUS‐NP and MGUS‐MM using a chi‐square test (Table 1), no significant differences between the median hemoglobin levels in MGUS‐NP (median 129 g/L, range 118‐140 g/L) and MGUS‐MM (median 128 g/L, range 115‐137 g/L) can be found (*P* = .36). Therefore, we cannot deduce that hemoglobin is a risk factor for this cohort.

### 3.6 Cumulative probability of progression

**FIGURE 1:**
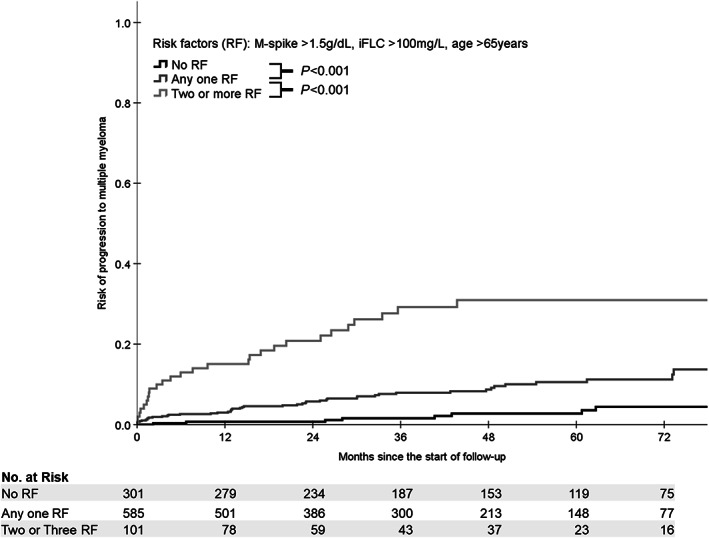
Impact of risk factors (RFs) on cumulative probability of progression from MGUS to MM. Probabilities were compared by log‐rank tests and is presented between groups. Absence of any risk factors were labeled as low risk (n = 301, 30%). Presence of only one RF, regardless of which, were labeled as intermediate risk (n = 585, 59%). Presence of any two, regardless of which, or all three RFs, were labeled as high risk (n = 101, 10%). Numbers below the graph indicate the number of patients within each risk group left at each time point. Significant differences of probabilities were observed between low risk and intermediate risk (*P* < .001), between low risk and intermediate risk (*P* < .001) and between intermediate and high risk (*P* < .001)

To further investigate the impact of the proposed risk factors, M‐spike >1.5 g/dL, age >65 years, and iFLC >100 mg/L, we stratified the patients according to the risk factors. As there was no significant difference, and the TTP‐KM curves were superimposed, between the groups with two and three risk factors, potentially due to the low number of patients with all the three risk factors (n = 21, 2%), these groups were merged. Patients were assessed as low risk (no risk factor, n = 301, 30%), intermediate‐risk (any one of the risk factors, n = 585, 59%), and high risk (two or more risk factors, n = 101, 10%). For each risk factor added, there was a clear and significant increase in the risk of progression. The five‐year cumulative probability of progression from MGUS to MM significantly correlated with the increasing number of risk factors, 2%, 11%, and 31% for low, intermediate, and high risk, respectively (Figure 1).

In the low‐risk patients, eight progressors were identified during the follow‐up, five of them with IgG and three with IgA MM. Five patients had abnormal FLCr at diagnosis (range 1.66‐13.2). Evolving M‐spike and iFLC was observed in only two of the eight patients before progression to MM.

### 3.7 Monitoring of risk factors

To explore whether an increase in heavy or light chain concentration during follow‐up, was associated with an increased risk of progression, further analyses were performed in patients with at least two sequential samples, 472 patients with MGUS‐NP and 44 patients with MGUS‐MM.

**FIGURE 2:**
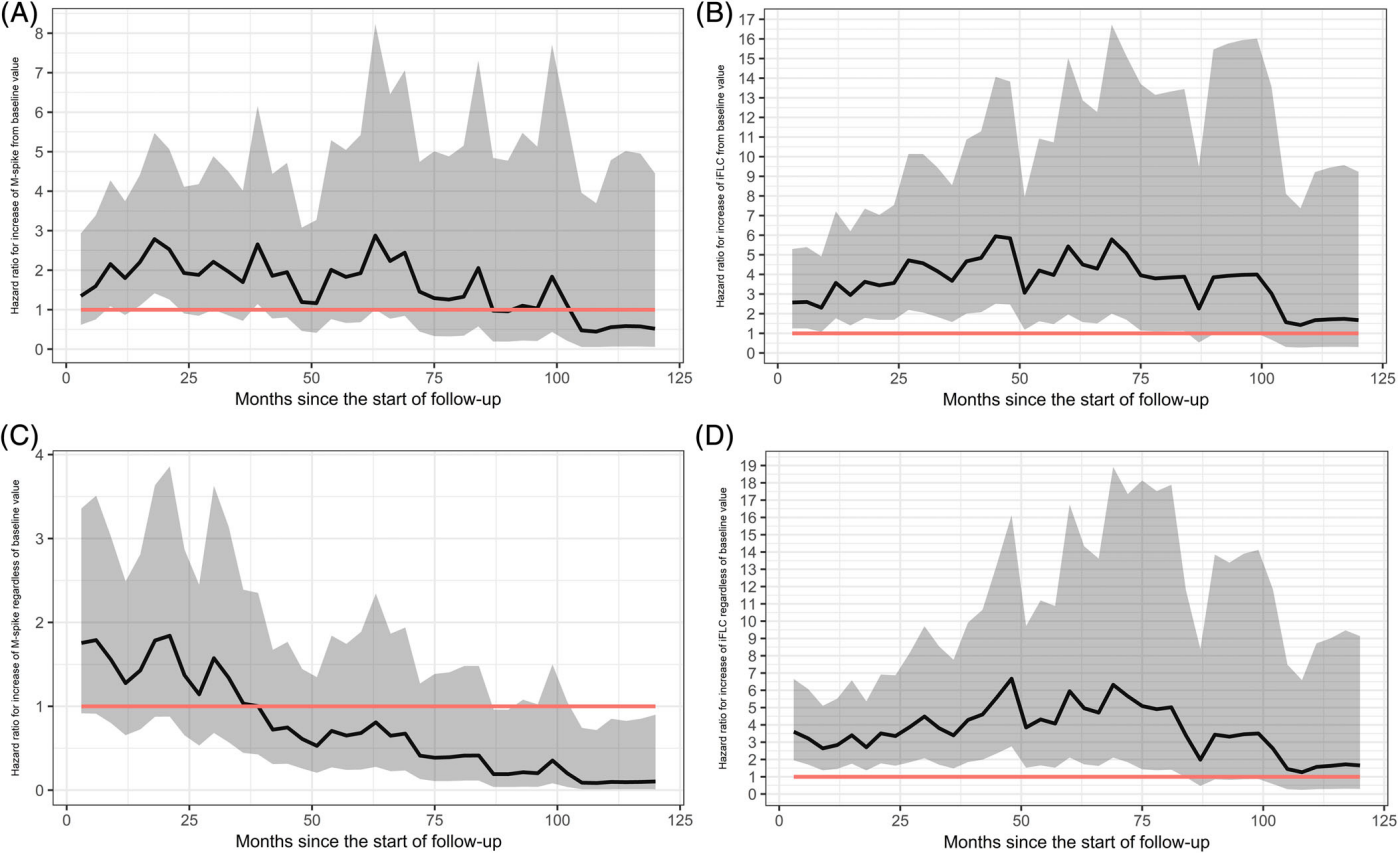
Risk of progression of MGUS to MM by evolving iFLC and M‐spike in plasma over 10 years of follow‐up. The Cox regression hazard ratios with the 95% confidence interval per 3 months’ time are plotted per graph. The black line represents HR, the gray area the 95% confidence interval, and the red line represents HR = 1. A, Absolute increase of 0.5 g/dL of serum M‐spike from baseline value. B, Absolute increase of 100 mg/L of plasma iFLC from baseline value. C, Increase in serum M‐spike >0.5 g/dL at any time during follow‐up regardless of baseline value. D, Increase in serum iFLC >100 mg/L at any time during follow‐up regardless of baseline value

An increase in M‐spike of >0.5 g/dL from baseline value was only significantly associated with increased risk of developing MM at three time periods during the first 5 years; at 9, 15 to 21 months and 30 months (Figure 2A). For the remainder of the follow‐up time, an increase in M‐protein >0.5 g/dL from baseline was not associated with significantly increased HRs. An increase in M‐spike of >0.5 g/dL, regardless of baseline values, was not associated with increased risk of progression to MM at any time point during the follow‐up time (Figure 2C).

An increase in iFLC of >100 mg/L, from baseline value, was consistently associated with a significantly increased risk of developing MM, 6 months from baseline, and up until 6.25 years, (Figure 2B). An increase in iFLC of >100 mg/L, regardless of baseline values, was significantly associated with higher risk of progression from baseline and up until 7 years, (Figure 2D).

To assess the performance of the evolving biomarkers as risk factors, we compared the area under the curve (AUC), sensitivity and specificity for the four factors (1‐increase of iFLC >100 mg/L from baseline, 2‐increase of iFLC >100 mg/L regardless of baseline, 3‐increase of M‐spike >0.5 g/dL from baseline, 4‐increase of iFLC >0.5 g/dL regardless of baseline). Increases in iFLC from baseline was the best predictor for risk of progression (AUC = 0.68; sensitivity 95%, specificity 33%).

## 4 DISCUSSION

In this study, we selected 4576 individuals out of which 987 were MGUS patients, with a median follow‐up of 64 months. Patients with MM, AL amyloidosis or other hematological malignancies were excluded. Since the progression of IgM MGUS in our material was exclusively to lymphoma or Waldenstrom's, all IgM MGUS patients were also excluded.

The large number of observations available for the sequential cohort enables us to evaluate the real‐world evolution of iFLC. However, the retrospective nature of the present study imposes limitations. As sFLC analyses were started in 2009 in Stockholm, almost half of the MGUS‐MM patients had no available results from before MM diagnoses and were therefore excluded. The lack of bone marrow examinations also limited our ability to evaluate other risk factors such as cytogenetics and flow cytometry.^18^ As bone marrow was assessed in only 73 out of 151 patients with M‐spike >1.5 g/dL, there is a risk that patients with SMM or MM could have been included. However, we could observe a similar proportion of patients progressing, 25% and 26%, in patients with and without bone marrow assessment at MGUS diagnosis. However, the probability of finding increased bone marrow plasma cells (≥10%) in low risk MGUS (M‐protein equal to or below 1.5 g/dL and IgG‐MGUS) is very low, 4.7%.^19^ Recently it was reported that omitting bone marrow assessment in patients with low risk MGUS without CRAB symptoms would result in a missed SMM or MM diagnoses in <1% of the patients.^20^

Various models to assess the risk of MGUS progression have been tested, with the Mayo Clinic model being incorporated into the current IMWG guidelines.^2^ From this model, based on an analysis of 1384 MGUS patients diagnosed between 1960 and 1994 in Minnesota, USA, the three risk factor, abnormal FLCr, non‐IgG isotype and M‐spike >1.5 g/dL, were identified.^5^, ^6^ Recent studies have not been able to confirm the increased risk of progression in non‐IgG (with or without IgM MGUS in the study population).^12^, ^13^, ^21^, ^22^ Similar to these publications, we have not seen a significantly increased risk of progression in the patient population with non‐IgG isotype. This difference might be due to; inclusion/exclusion of IgM MGUS in the study populations and the differences in follow‐up time. However, the inconsistency of the non‐IgG isotype renders it unreliable as a risk prediction factor.

Beside the already established risk factors recommended by the IMWG, other groups have suggested that immunoparesis,^10^, ^12^, ^13^, ^18^ aberrant plasma cells in bone marrow plasma cell compartment^18^ and aneuploidy^18^ to be associated with risk of progression. In our cohort, two other factors emerged as highly significant risk factors for progression, age and iFLC concentrations at baseline. Age was an independent risk factor for progression in both uni‐ and multivariate analyses, as continuous factor and with different cut‐offs. Patients >65 years old showed the highest probability of progression to MM (HR 2.1, 95% CI 1.31‐2.40, *P* = .002). Similar findings were previously reported.^12^

Knowing that the incidence of abnormal FLCr is more common in MM (>95%) compared to MGUS (30‐42%),^4^, ^11^ we aimed attesting our hypothesis that evolving iFLC is the major factor for progression from MGUS to MM status, and therefore included sequential samples in the current database. The hazard ratio for evolving iFLC from baseline as a predictor of progression was consistently increasing from 6 months until 6.25 years of follow‐up, hence the hypothesis was confirmed. It is conceivable that the HR would either remain high or increase with time beyond 6 years. We could also show that an increase of iFLC of >100 mg/L, regardless of baseline value, was significant up to 7 years. The results show that evolving iFLC levels after MGUS diagnosis is an important predictor of developing MM. We therefore recommend that iFLC should be included in the monitoring of MGUS patients.

Abnormal FLCr can be observed already at low tumor burden with low production of M‐protein. Thus, abnormal FLCr could potentially be observed in a large proportion of the MGUS populations; we observed in our study that abnormal FLCr was present at diagnosis in 397 (40%) patients, which is similar to the 33%‐47% observed in the previous studies.^6^, ^12^, ^13^ One could argue that a risk factor shown in such a large proportion would make it unsuitable for distinguishing between high risk and low‐risk patients. The incorporation of iFLC >100 mg/L in our prediction model leads to loss of significance of FLCr, most likely as iFLC >100 mg/L will in the majority of the patients give an abnormal FLCr, in our cohort two (2%) out of 125 patients with iFLC >100 mg/L had a normal FLCr. According to our findings, iFLC >100 mg/L, M‐spike >1.5 g/dL, and age >65 years should be incorporated in the risk stratification of progression of MGUS to MM. In an attempt to further enhance the risk stratification, we suggest utilizing all three risk factors by grouping them from low risk (no risk factors), intermediate (one risk factor), and high risk (two and three risk factors).

Patients with low risk (30%) had a very low five‐year risk to progression (2%). Only eight (3%) of the patients without any risk factor progressed during follow‐up in our cohort. Thus, we suggest that patients with low risk of progression could be emitted from follow‐up. The five‐year risk of progression observed in intermediate‐risk was 11% compared to 31% in high‐risk patients. On the contrary to low‐risk patients, the increased risk observed in both intermediate and high‐risk patients supports these patients’ regular monitoring. As evolving iFLC seems to be a significant factor during follow‐up, we suggest every 6‐12 months, for intermediate‐risk, and quarterly, for high risk, monitoring with iFLC during the first 5 years.

The main goals of this study were to incorporate the iFLC into prognostic models and the monitoring of MGUS patients. The presence of evolving iFLC is a clinically significant risk factor of MGUS progression. Based on the data reported here we propose to monitor iFLC for patients with evolving iFLC values >100 mg/L quarterly. We further recommend that incorporation of M‐spike >1.5 g/dL, age >65 and iFLC >100 mg/L as risk factors in prediction models.

## AUTHOR CONTRIBUTIONS

Charlotte Gran and Hareth Nahi conceived the study and oversaw overall direction and planning. Charlotte Gran and Hareth Nahi wrote the manuscript with input from all authors. Johan Liwing, Andre Verhoek and Ana Gezin analyzed the data. Evren Alici and Hareth Nahi supervised the project. All authors critically revised the manuscript and approved the final version.

## Supporting information


**Appendix S1:** Supporting Information

## Data Availability

The data that support the findings of this study are available upon reasonable request from the corresponding author. The data are not publicly available due to privacy or ethical restrictions.
