# Supplementary material for: Comparative evaluation of involved free light chain and monoclonal spike as markers for progression from monoclonal gammopathy of undetermined significance to multiple myeloma
Source: Am J Hematol. 2020 Sep 29;96(1):23–30. doi: 10.1002/ajh.25999 (PMC7756706; doi:10.1002/ajh.25999)

Table 1 supplemental

Patient Characteristics for sequential cohort. Percentages may not sum up to 100 due to rounding. MGUS-NP denotes MGUS patients that does not progress to MM, MGUS-MM MGUS patients that progress to MM, M-spike concentration of M-component, iFLC involved FLC, sFLC serum FLC, and ISS Multiple Myeloma International Staging System.

Fig 1 supplemental

Overview of the database utilized in the analysis of prognostic factors for progression of MGUS to MM. The study population includes individuals older than 18 years, with an analysis of serum or urine electrophoresis (s/uPEP) within 7 days of sFLC measurement were included. In total, 4756 individuals were included, which generated 30,052 sampling occasions from all individuals during the study period. Individuals that had no plasma cell disorders nor evidence of M-protein in s/uPEP or abnormal sFLC ratio were excluded as were patients with diagnoses of either plasma cell disorders or hematological diseases other than MGUS as well as IgM MGUS.


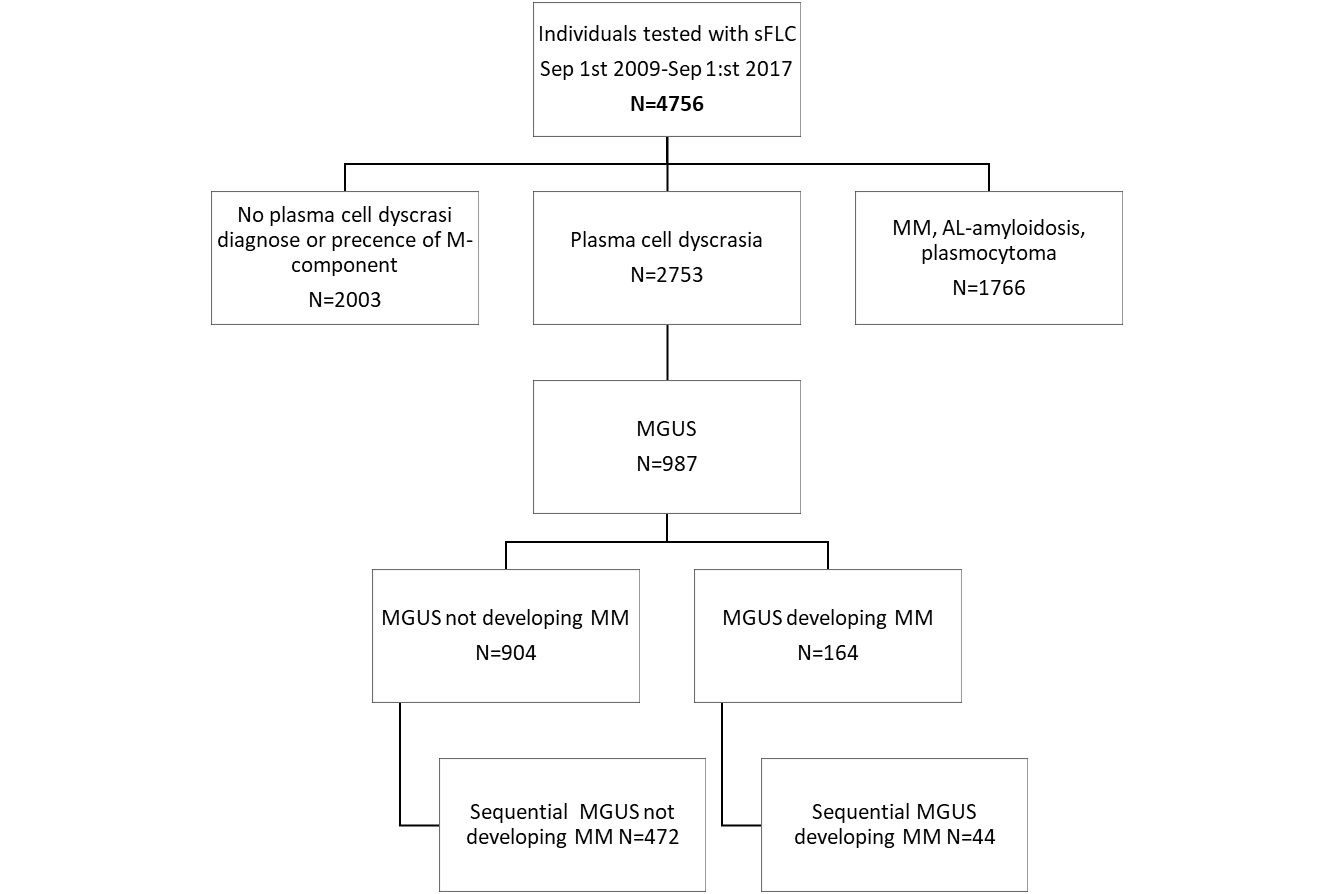


Fig 2 supplemental

Cumulative probability of progression from MGUS to MM shown as number of patients progressing per 6 months interval.


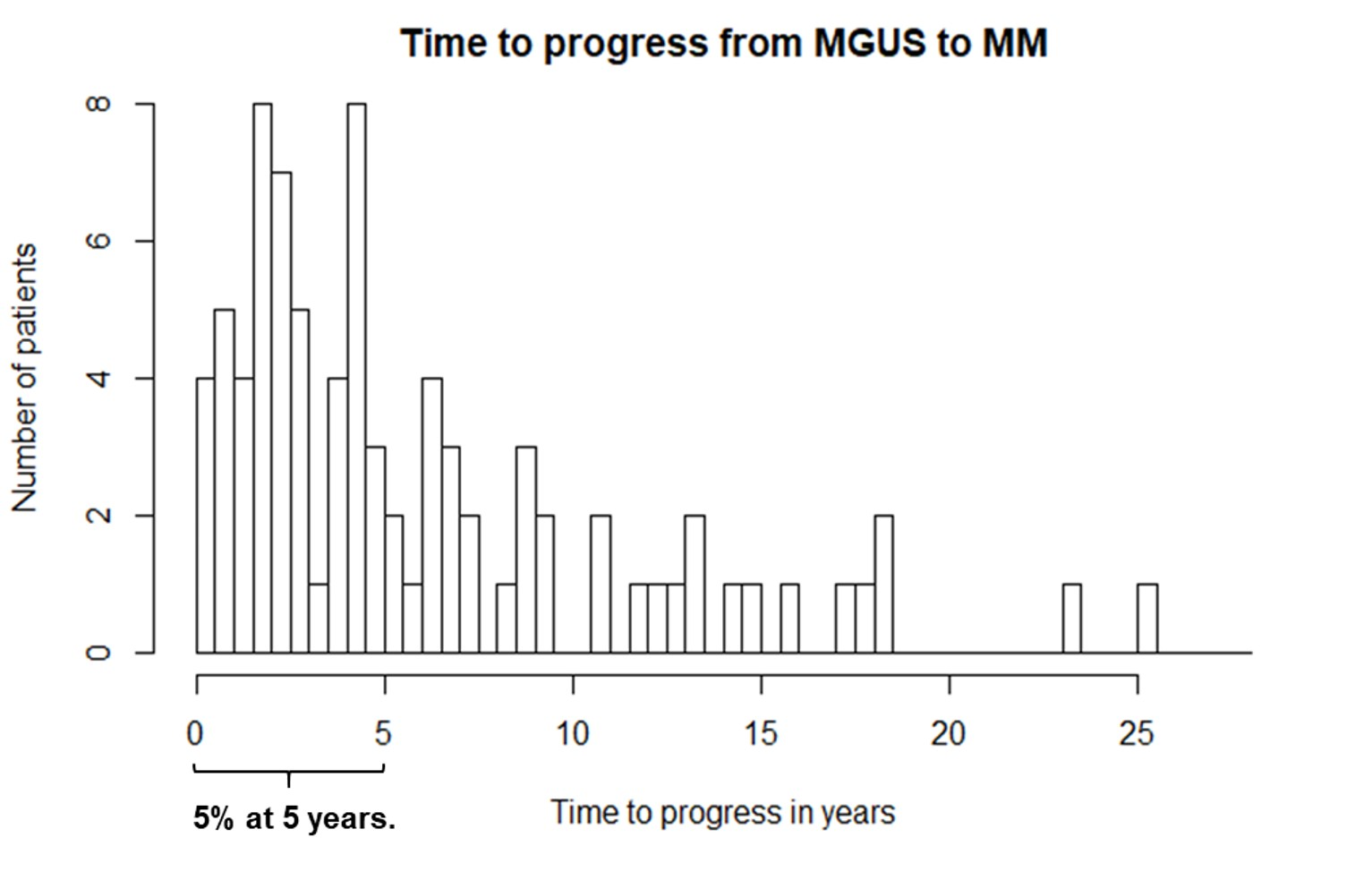

Supplement: Supplementary file 1 — Appendix S1: Supporting Information [file AJH-96-23-s001.docx]
